# Love, culture, rationality, and learning in repeated games

**DOI:** 10.3389/fsoc.2025.1515473

**Published:** 2025-10-31

**Authors:** Andrés Cendales, Luis Eduardo López-Montenegro, Hugo Guerrero-Sierra

**Affiliations:** ^1^Departamento de Economía y Administración, Facultad de Ciencias Jurídicas y Sociales, Universidad de Caldas, Manizales, Colombia; ^2^Departamento de Matemáticas, Facultad de Ciencias Exactas y Naturales, Universidad de Caldas, Manizales, Colombia; ^3^Facultad de Relaciones Internacionales, Estrategia y Seguridad, Universidad Militar Nueva Granada, Cajicá, Colombia

**Keywords:** love, culture, rationality, learning, repeated games, computational simulation, game theory

## Abstract

The dynamics within romantic relationships reflect and influence broader family structures. This article examines family dynamics by analyzing various social identities and power structures, such as gender and social class, through a rational choice methodological approach. It challenges the notion that past family stability was due to stronger values, arguing instead that it was supported by oppressive structures, primarily patriarchal. By critiquing these power structures, we aim to present a more inclusive and equitable perspective on family dynamics. We introduce a rational choice model from game theory to analyze stability patterns in romantic relationships, focusing on empathy coefficients and cultural factors. This model investigates the role of economic altruism theory and cultural identities in shaping empathy and their impact on relationships. The study also explores individual wellbeing across different cultural contexts, including representations of courtly love and patriarchal norms. An instrumental rationality perspective is proposed to address the irrationality of love from a rational standpoint in family literature. Simulation is crucial for validating the model and evaluating the assumptions in realistic contexts. It provides insights into the model's behavior under various conditions, highlighting limitations and aspects previously unconsidered. This approach is essential for verifying implementation, validating assumptions, and exploring data uncertainties. By employing this methodology, future research can gain a deeper understanding of the complexities in family dynamics and romantic relationships.

## 1 Introduction

The dynamics and experiences within romantic relationships reflect and affect broader family dynamics. Given that the values, behaviors, and roles established in a romantic relationship can influence the structure and functioning of the family, we propose an analysis of family dynamics by considering various social identities and power structures (such as gender and social class) using a rational choice methodological approach (an economic perspective). The way couples make decisions regarding mutual care determines the family experience. We aim to challenge the dominant narrative that families in the past were more stable due to stronger values. We assert that families in the past were more stable because those values were supported by structures of oppression (primarily patriarchal). In addition to questioning the power structures and traditional narratives that have been used to define and understand families, we promote a more inclusive and equitable vision.

This article introduces into the literature on family theory a rational choice model that characterizes the romantic relationship between two individuals within game theory to examine stability patterns in the relationship based on the values of their empathy coefficients (Duck, 1982; Wood, 1982; Knapp, 1984). The Economic theory of altruism assumes that if an individual's utility depends on the utility of others, positive interdependence can precisely be termed as love (Bolle, 1992; Yan et al., 2022; Watkins et al., 2022). While it is true that individuals derive (altruistic) benefits from the wellbeing of their partner, the magnitude and nature of these benefits vary depending on the degree of reciprocity and empathy^1^ (Veblen, 1994; Duesenberry, 1949). Empathy is a parameter that involves emotional connection and fosters not only effective communication and conflict resolution but also the cultivation of intimacy, among other key features in the stability of a romantic relationship.

The empathy coefficient of each individual in a romantic relationship depends on their cultural identity regarding love, as love is influenced by social, economic, or religious considerations^2^ (Heshmati and Oravecz, 2022). The theoretical model we propose allows for the consideration of different empathy coefficients that individuals experience for their partners based on their cultural identities regarding love. In each case, the payoffs individuals receive in relationships differ. The (qualitative) payoff a woman obtains if her cultural identity of love is patriarchal, involving submission and an unequal power position, is very different from the payoff the woman obtains if her cultural identity of love is hedonistic within the context of a liberal society, where there is greater freedom from the constraints imposed by the patriarchal man. This is consistent with various studies that emphasize the relative nature of individual happiness or wellbeing, including (Easterlin 1974); (Frank 1985, 1989); (Clark and Oswald 1996); (Ray and Robson 2012), and many others (see Ray and Vohra, 2020). The study of romantic relationships from an instrumental rationality perspective has already seen some exploration (Bear and Rand, 2019; Baumeister et al., 2017).

The literature developed in family theory has explored the potential relevance of chaos theory to understand the development and change in romantic relationships precisely due to the seemingly illogical nature of love, which results in relationships being able to change quickly and spontaneously, making predicting their trajectory quite problematic (Koopmans, 1998; Gottman, 1991; Barton, 1994; Weigel and Murray, 2000). In the opening act of the narrative “Symposion,” specifically in the *Praise of Platonic Love*, (Kundera 1980), through the conversation between Flajsman and Alzbeta, expresses the idea that “love is precisely that which lacks logic” (Kundera, 1980, p. 54). This assertion suggests that in the realm of love, an individual's behavior often defies logic and reason. If love involves behaviors that are not rational, does this preclude explaining these behaviors from an instrumental rationality perspective? Not necessarily.^3^ The objective of this article is to utilize rational choice theory to elucidate various behaviors associated with different conceptions of love. One of these conceptions is courtly love, portrayed in erotic literature within the theatricality of love, while others are linked to behaviors observed in certain cultures and time periods. A central aim of this article is to reconcile the divergent perspectives that often characterize discussions about love, a recurring theme in culture. Since love is often described as illogical, while rational choice theory posits a particular conception of rationality, the question arises regarding the degree of rationality present in people's various conceptions of love, which are rife with contradictions and suboptimal outcomes. Each conception of love implies its own logic in relation to the goals, desires, and whims of those involved. There is a certain underlying rationality in each conception of love, allowing us to discern aspects of its perplexing logic that may initially appear illogical.

The article is structured as follows. Section 2 presents a brief bibliometric review of how the literature has grown according to thematic nodes and areas of knowledge to demonstrate the increasing importance of the proposed discussion in this article. Section 3 presents the theoretical model, and Section 4 describes the results of the characterization. In the results, we present the simulation of the theoretical model for each conception of love. The simulation provides an opportunity to validate that the implementation adheres to the original model and produces coherent results. Additionally, the simulation allows for an evaluation of the validity of the assumptions of the theoretical model by examining how the model behaves in a more realistic context. The simulation can reveal limitations or previously unconsidered aspects. More precisely, with the simulation, specific cases or particular situations are addressed to explore and examine these cases, providing a more detailed and concrete understanding of how the model behaves under different conditions. The simulation provides a valuable tool for verifying implementation, validating assumptions, exploring untested cases, and accounting for uncertainty in the data. Finally, Section 5 provides the concluding remarks.

## 2 The theoretical model

Let *G* be a static game of complete information with two players. We can consider the set of actions available to each player as their respective strategy space. Let *S*_*i*_ = {**a**, **b**} denote the set of life projects from which individual *i* ∈ *I* = {1, 2} can choose, such that *S*_1_ = *S*_2_. Let *u*_*i*_:*S*_*i*_ → [0, 1] be the utility function of individual *i* ∈ *I* that represents their preference relation ≻_*i*_:*S*_*i*_→*S*_*i*_, where ≻_*i*_ is complete. For any project **x** ∈ *S*_*i*_, let *u*_*i*_(**x**) ∈ [0, 1] denote the extent to which individual *i* ∈ *I* identifies with the project **x**. Without loss of generality, we assume that *u*_*i*_(**a**)+*u*_*i*_(**b**) = 1 for each *i* ∈ *I*. It is further assumed that *u*_1_(**a**)≥*u*_1_(**b**) and *u*_2_(**b**)≥*u*_2_(**a**), implying that player 1 strictly prefers project **a** to project **b**, and player 2 strictly prefers project **b** to project **a**. We can verify that $u_{1}\left(\text{a}\right)\geq \frac{1}{2}\geq u_{1}\left(\text{b}\right)$ and $u_{2}\left(\text{b}\right)\geq \frac{1}{2}\geq u_{2}\left(\text{a}\right)$. Let *u*_1_ = *u*_1_(**a**)−*u*_1_(**b**) and *u*_2_ = *u*_2_(**b**)−*u*_2_(**a**) be the identity biases in each case. We define the magnitude of inclination or empathy of individual *i* toward the life project z ∈ *S*_−*i*_ most preferred by individual −*i* as *k*_*i*_ ∈ (*u*_*i*_/*u*_−*i*_(z), +∞). Let ***u***_*i*_:*S*_*i*_×*S*_−*i*_ → ℝ be the payoff function of player *i* such that


$$u_{i}(x,y)=\{\begin{array}{l} \begin{array}{ccc} u_{i}(x)+k_{i}\cdot u_{-i}(y) & \text{if} & x=y\\ u_{i}(x) & \text{if} & x\neq y \end{array} \end{array}$$

In the event that the players fail to reach a consensus on a project, player *i*'s payment will be based solely on their personal identification with the chosen project. However, if the players do agree on a life project denoted by **x**, player *i* will not only experience their own identification *u*_*i*_(**x**) with project **x**, but will also perceive a certain magnitude *k*_*i*_·*u*_−*i*_(**y**) of the identification *u*_−*i*_(**y**) that player −*i* has with their own project **y**, based on player *i*'s level of empathy or affection *k*_*i*_ toward player −*i*. The degree to which player *i* perceives player −*i*'s happiness as their own is determined by the value of *k*_*i*_. Consequently, if *k*_*i*_ tends to infinity, player *i* has fallen in love with player −*i*, and therefore sublimates or idealizes player −*i*, resulting in player *i*'s happiness being intrinsically tied to that of player −*i*.

Let ***u***_*i*_:*S*_1_×*S*_2_ → ℝ be the payoff function of player *i* = 1, 2 such that


$$M_{i}=\left(\begin{array}{cc} u_{i}(a,a) & u_{i}(a,b)\\ u_{i}(b,a) & u_{i}(b,b) \end{array}\right)$$

is the payoff matrix. Given the payoff matrices *M*_1_ and *M*_2_, we have the payoff bimatrix


$$\begin{array}{l} \left(M_{1},M_{2}\right)=\left(\begin{array}{cc} u_{1}\left(\text{a}\right)+k_{1}\cdot u_{2}\left(\text{a}\right),u_{2}\left(\text{a}\right)+k_{2}\cdot u_{1}\left(\text{a}\right) & u_{1}\left(\text{a}\right),u_{2}\left(\text{b}\right)\\ u_{1}\left(\text{b}\right),u_{2}\left(\text{a}\right) & u_{1}\left(\text{b}\right)+k_{1}\cdot u_{2}\left(\text{b}\right),u_{2}\left(\text{b}\right)+k_{2}\cdot u_{1}\left(\text{b}\right) \end{array}\right) \end{array}$$

We say that *G* = [{*S*_1_, *S*_2_}, {***u***_1_, ***u***_2_}] is the game of love. We shall denote the *j*−th column of matrix *M*_*i*_ as *M*_*i*:*j*_, and we shall denote the *j*−th row of matrix *M*_*i*_ as *M*_*ij*:_.

## 3 Characterization results

Lemma 1. *Fictitious Play Property* — Following (Miyasawa 1961) and (Monderer and Shapley 1996), we can state that *G* has the fictitious play property if *G* is a non-degenerate game.

*Proof*. In effect,

*u*_1_(a) + *k*_1_·*u*_2_(a) − *u*_1_(a) − *u*_1_(b) + *u*_1_(b) + *k*_1_·*u*_2_(b) = *k*_1_·*u*_2_(a) + *k*_1_·*u*_2_(b) = *k*_1_>0

and

*u*_2_(a) + *k*_2_·*u*_1_(a) − *u*_2_(b) − *u*_2_(a) + *u*_2_(b) + *k*_2_·*u*_1_(b) = *k*_2_·*u*_1_(a) + *k*_2_·*u*_1_(b) = *k*_2_>0

Let us assume for a moment that *k*_1_ = *u*_1_/*u*_2_(b). Under this assumption, it can be concluded that individual 1 does not experience any empathy, affection, or love toward individual 2. Let ***u***_1_:*A*_1_×*A*_2_ → ℝ be the payoff function of player 1 such that *k*_1_ = *u*_1_/*u*_2_(b). The payoff matrix for this function is as follows:


$$M_{1}=\left(\begin{array}{cc} u_{1}(a,a) & u_{1}(a,b)\\ u_{1}(b,a) & u_{1}(b,b) \end{array}\right)=\left(\begin{array}{cc} u_{1}(a)+u_{1}\cdot \frac{u_{2}(a)}{u_{2}(b)} & u_{1}(a)\\ u_{1}(b) & u_{1}(a) \end{array}\right)$$

In this possible world, if player 2 chooses project **b**, player 1 remains indifferent as to whether or not it coincides with player 2, unless player 2 chooses project **a**. In the latter case, player 1 strictly prefers project **a**, which is precisely his most preferred project. Likewise, if we define the coefficient of empathy or love of individual 2 by individual 1 as *k*_2_ = *u*_2_/*u*_1_(a), then we can infer that individual 2 does not experience any empathy, affection, or love for individual 1. Thus, if *k*_*i*_ = *u*_*i*_/*u*_−*i*_(z) such that z ∈ *S*_−*i*_ represents the most preferred project of player −*i*, then player *i* exhibits a complete absence of love, affection, or inclination toward player −*i*.

Theorem 2. Let *G* = [{*S*_1_, *S*_2_}, {***u***_1_, ***u***_2_}] be the game of love. Let


(1) $$\begin{array}{l} △\left(S_{1}\right)=\left\{\sigma_{i}^{⊤}=\left(\sigma_{i}\left(\text{a}\right),\sigma_{i}\left(\text{b}\right)\right):\sigma_{i}\left(\text{x}\right)\geq 0\text{ for each }\text{x}\in S_{i}\text{ and }\sigma_{i}\left(\text{a}\right)\text{+}\sigma_{i}\left(\text{b}\right)=1\right\} \end{array}$$

be the simplex of *S*_*i*_ such that σ_1_ = p and σ_2_ = q where


(2) $$\begin{array}{l} \left(\sigma_{1}\left(\text{a}\right),\sigma_{1}\left(\text{b}\right)\right)=\left(p,1-p\right) \end{array}$$

and


(3) $$\begin{array}{l} \left(\sigma_{2}\left(\text{a}\right),\sigma_{2}\left(\text{b}\right)\right)=\left(q,1-q\right) \end{array}$$

The Nash equilibria of the game *G* = [{*S*_1_, *S*_2_}, {***u***_1_, ***u***_2_}] are


(4) $$\begin{array}{l} {\left({\text{p}}^{*},{\text{q}}^{*}\right)}^{⊤}=\left(\left(1,0\right),\left(1,0\right)\right) \end{array}$$

(5) $$\begin{array}{l} {\left({\text{p}}^{*},{\text{q}}^{*}\right)}^{⊤}=\left(\left(0,1\right),\left(0,1\right)\right) \end{array}$$

and


(6) $$\begin{array}{l} \left({\text{p}}^{*},{\text{q}}^{*}\right)=\left(\left(u_{1}\left(\text{b}\right)+u_{2}/k_{2},u_{1}\left(\text{a}\right)-u_{2}/k_{2}\right),\left(u_{2}\left(\text{b}\right)-u_{1}/k_{1},u_{2}\left(\text{a}\right)+u_{1}/k_{1}\right)\right) \end{array}$$

*Proof*. Let *v*_*i*_:△(*S*_1_) × △(*S*_2_) → ℝ be the expected payoff function of player *i* = 1, 2 such that


(7) $$\begin{array}{l} v_{i}\left(\text{p},\text{q}\right)=\left(p,1-p\right)\cdot M_{i}\cdot \left(\begin{array}{c} q\\ 1-q \end{array}\right) \end{array}$$

It is trivially verified that


(8) $$\begin{array}{l} \frac{\partial v_{1}\left(\text{p},\text{q}\right)}{\partial p}=q\cdot k_{1}+u_{1}-k_{1}\cdot u_{2}\left(\text{b}\right) \end{array}$$

On the other hand, it is trivially verified that


(9) $$\begin{array}{l} \frac{\partial v_{2}\left(\text{p},\text{q}\right)}{\partial q}=p\cdot k_{2}-u_{2}-k_{2}\cdot u_{1}\left(\text{b}\right) \end{array}$$

From expressions (8) and (9) it follows that


(10) $$\begin{array}{l} \left({\text{p}}^{*},{\text{q}}^{*}\right)=\left(\left(u_{1}\left(\text{b}\right)+\frac{u_{2}}{k_{2}},u_{1}\left(\text{a}\right)-\frac{u_{2}}{k_{2}}\right),\left(u_{2}\left(\text{b}\right)-\frac{u_{1}}{k_{1}},u_{2}\left(\text{a}\right)+\frac{u_{1}}{k_{1}}\right)\right) \end{array}$$

is the Nash equilibrium in mixed strategies (NEMS) of the game *G*. Additionally, given that *k*_*i*_≥*u*_*i*_/*u*_−*i*_(z) it is trivially verified that


(11) $$BR_{i}\left(\sigma_{-i}\right)=\{\begin{array}{l} \begin{array}{ccc} \left\{e_{1}\right\} & \text{if} & \sigma_{-i}=e_{1}\\ \left\{e_{2}\right\} & \text{if} & \sigma_{-i}=e_{2} \end{array} \end{array}$$

for each *i* = 1, 2 such that {*e*_1_, *e*_2_} is the canonical basis of ℝ^2^. Hence,


$$\left({\text{p}}^{*},{\text{q}}^{*}\right)=\left(\left(1,0\right),\left(1,0\right)\right)$$

and


$$\left({\text{p}}^{*},{\text{q}}^{*}\right)=\left(\left(0,1\right),\left(0,1\right)\right)$$

are the Nash equilibria in pure strategies.

### 3.1 *Of courtly love* (L'amour courtois)

The narratives of tragic courtly love, as one of the approaches within courtly love literature, overwhelmingly exhibit a tragic denouement. These are accounts of impossible and heart-rending loves, laden with insurmountable obstacles that lead to the unhappiness and death of the lovers, *i.e*., to a profound discord. One of the already classic pieces of modern literature that reenacts the conception of courtly love is proffered by Oriana Fallaci:

(...) *The necessity of love is a yearning that must find fulfillment within a union, yet its quantity or quality is scarcely ever harmoniously balanced between the two, by symmetry and synchrony: when he is available, she is not; when she is available, he is not... Or perhaps they are both available, yet to satiate his need requires but a sip, whereas to quench her yearning, a river's flow would not suffice, and vice versa. In my contemplation, the anathema God cast upon Adam and Eve upon their expulsion from the Terrestrial Paradise was not “In pain shall you bring forth children, and your husband shall rule over you” or “By the sweat of your brow you shall eat bread.” It was rather: “When he desires you, you shall not desire him; when she desires you, you shall not desire her”*. (Fallaci, 1992)

The separation, betrayal, or ultimate tragedy constitutes the denouement of the narratives despite the devotion and passion of their lovers. Certain classic tales within courtly love literature encompass Tristan and Isolde, The Death of Arthur, and Romeo and Juliet. The story of La Celestina, attributed to Fernando de Rojas, provides an example of this phenomenon. (Marías 2015) aptly describes the first moment in which Calisto, in a poetic display, confesses to having fallen in love with Melibea:

*When Calisto falls in love with Melibea and is rejected by her, what happens? His servants, Parmeno and Sempronio, suggest he contact Celestina. Celestina is a technician; you call Celestina like you call an electrician or a plumber, and what techniques she has! She has her persuasion techniques... There is a particularly interesting text where Calisto exaggerates, saying that Melibea is his God. His servant tells him, “but you are not a Christian.” Calisto responds, “I, Christian? I am a Melibean, I adore Melibea, I believe in Melibea, and I love Melibea.” “I am a Melibean...” (...) This text in which Calisto says “I am a Melibean, I adore Melibea, I believe in Melibea, and I love Melibea” is precisely the exaggerated, extreme, vehement, passionate expression of that unique love because Melibea has become his project - this is the characteristic*. (Marías, 2015, p. 4)

The love between Calisto and Melibea can be represented mathematically by the terms *k*_1_ and *k*_2_, where *k*_1_ tends to infinity, assuming Calisto as the row player, and *k*_2_ tends to infinity, assuming Melibea as the column player. These terms signify the degree of love, affection, or inclination of each toward the other. In courtly love, as noted from a psychoanalytic perspective, there exists an obsessive overvaluation of the beloved object (Koenigsberg, 1967). The ultimate outcome of their love story is death, which befalls both of them. Death is often portrayed in other cases through mutual disdain, weariness, or by simply acquiring oblivion through the art of forgetting, as (Borges 1999) reminded us in “*the world is no longer magical*”. The Calisto's best response correspondence

**Figure 1:**
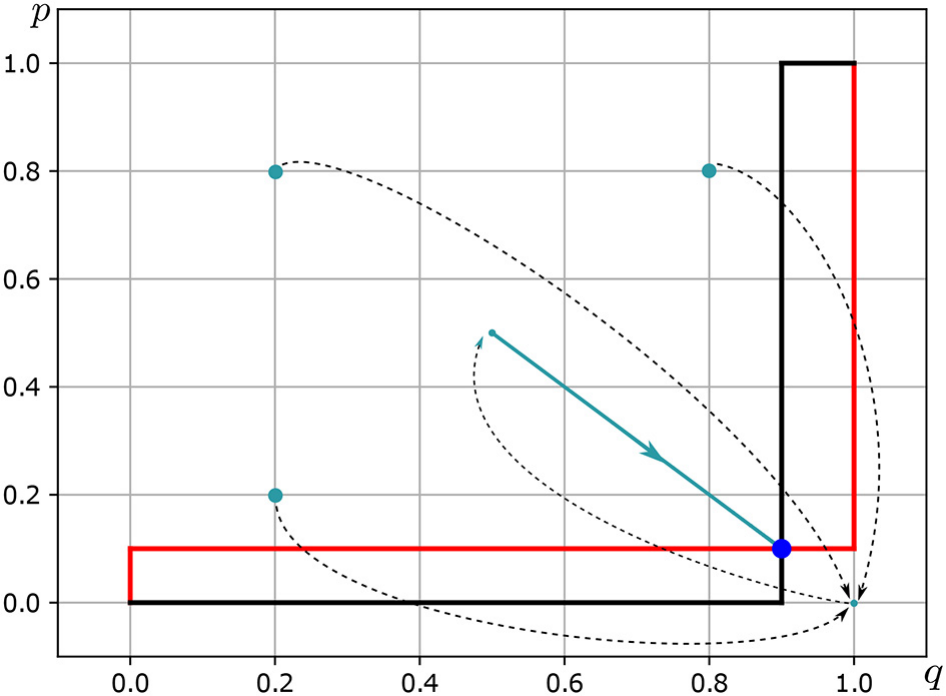
Nash equilibrium in mixed strategies *Of Courtly Love*. Source: Own elaboration.

*BR*_1_:△(*S*_2_)⇉△(*S*_1_) is as follows (see Figure 1—Red line):


$$BR_{1}\left(q\right)=\{\begin{array}{l} \begin{array}{ccc} \left\{p^{\text{*}}:{\text{p}}^{\text{*}}\text{=0}\right\} & \text{if} & q<u_{2}\left(b\right)\\ \left\{p^{\text{*}}:\text{0}\leq \text{p}\leq \text{1}\right\} & \text{if} & q=u_{2}\left(b\right)\\ \left\{p^{\text{*}}:{\text{p}}^{\text{*}}\text{=1}\right\} & \text{if} & q>u_{2}\left(b\right) \end{array} \end{array}$$

The Melibea 's best response correspondence

*BR*_2_:△(*S*_1_) ⇉△(*S*_2_) is as follows (see Figure 1—Black line):


$$BR_{2}\left(p\right)=\{\begin{array}{l} \begin{array}{ccc} \left\{q^{\text{*}}:{\text{q}}^{\text{*}}\text{=0}\right\} & \text{if} & p<u_{1}\left(b\right)\\ \left\{q^{\text{*}}:\text{0}\leq \text{p}\leq \text{1}\right\} & \text{if} & p=u_{1}\left(b\right)\\ \left\{q^{\text{*}}:{\text{p}}^{\text{*}}\text{=1}\right\} & \text{if} & p>u_{1}\left(b\right) \end{array} \end{array}$$

In consequence, ${\left(\text{p},\text{q}\right)}^{⊤}=\left(\left(u_{1}\left(\text{b}\right),u_{1}\left(\text{a}\right)\right),\left(u_{2}\left(\text{b}\right),u_{2}\left(\text{a}\right)\right)\right)$ is the NEMS of the game *of courtly love*.

In this equilibrium, Calisto exhibits a peculiar behavior in which he chooses his preferred project, a, with a low intensity (probability) *u*_1_(b) ∈ [0, 1/2), while also choosing project b, the least preferred by him but the most preferred by Melibea, with a high intensity (probability) *u*_1_(a) ∈ (1/2, 1]. In other words, Melibea has become Calisto's project. A similar phenomenon occurs in the case of Melibea. At this point, (Marías 2015) asserts,

*What is love? I think ninety people would start by saying “love is a feeling that...” and then they would go on to say different things. I believe this is not true: love is not a feeling. Love is obviously accompanied by loving feelings: there are many loving feelings that are concomitant, that accompany love. But love is not a feeling: it is a personal situation that alters a person's reality. It is not merely a feeling. It is obvious that it is accompanied by feelings, which are expressed, manifested in feelings that can be variable, but it is not fundamentally a feeling: it is a change in personal reality. Love alters a person's reality, turns them into something different and, therefore, is a phenomenon deeper than purely sentimental—although, I repeat, it is inseparable from it*. (Marías, 2015, p. 1)

According to (Marías 2015), Calisto has projected himself onto Melibea by taking her project as his own and has fallen into the intense alienation that characterizes love. As a result, his personal reality has been transformed. Similarly, Melibea selects project b—her preferred project—with a low intensity (probability) *u*_2_(a) ∈ [0, 1/2], while she chooses project a—the project Calisto identifies with most—with a high intensity (probability) *u*_2_(b) ∈ (1/2, 1]. In consequence, Calisto seeks Melibea in her most preferred project, with Melibea being his project, and Melibea looks for Calisto in his most preferred project, with Calisto being her project. The outcome of their pursuit is absurd: Calisto and Melibea never meet, despite searching for one another. Isn't this ludicrous?

Let us examine a story from the 20th century—*Eduard and God* in *The Book of Ridiculous Loves* by (Kundera 1980). This story is intriguing as it exposes the proximity between a deliberately designed and executed strategy and the ensuing alienation experienced by the subject of said strategy, even if this state of alienation is ephemeral or temporary.

According to the story, Eduard is engaged in two projects. Firstly, project a pertains to mundane love, which is purely sexual and driven toward achieving coital pleasures. Eduard actively seeks out flirtations that eventually lead to fornication. Secondly, project b is related to his love for God and his faith, which is not his preferred project, given that he is part of the Stalinist revolutionary youth and does not believe in God. Project a is so closely associated with worldly and carnal love that its identity *u*_1_(a) converges to 1, while project b is so little identified with love for God and faith *u*_1_(b) that it converges to 0. In tragicomedy, the presence of male and female forms the basis of lyricism. Alice, a character in the story, is portrayed as a deeply chaste, decent, and fervent believer in God.

Her love for God is of utmost importance to her and is strengthened by a strong ethical foundation based on the Catholic commandments. In contrast, her worldly love project cannot be her highest priority. As a result, Alice's identity with the worldly love project *u*_2_(a) converges to 0, while her identity with the God-loving project *u*_2_(b) converges to 1. Eduard realizes that following his encounter with Alice, he needs to adopt a strategy of imitating a fervent believer in God:

*Until this time it had never occurred to him to believe in God. He understood, however, that he must not admit this. On the contrary, he saw that he should take advantage of the opportunity and knock together from faith in God a nice Trojan horse, within whose belly, according to the ancient example, he would slip into the girl's heart unobserved. Only it wasn't so easy for Eduard simply to say to Alice, “I believe in God”; he wasn't at all impudent, and he was ashamed to lie; the simplicity of lying repelled him; if a lie was absolutely necessary, he wanted it to remain as close as possible to the truth*. (Kundera, 1980, p. 109)

Having faith in Eduard's apparent commitment, Alice reciprocates by revealing her intentions:

*I would like to teach you to love him (God Antifornicator) just as I do*. (Kundera, 1980, p. 110)

Eduard must engage in a battle of deterrence against Alice's love for God, specifically the God Antifornicator, while also attempting to dissuade her from pursuing worldly love. Similarly, Alice will consciously and deliberately try to discourage Eduard from embracing the project of love for God, the God Antifornicator, while also discouraging him from pursuing worldly love.

*This God embodied a single idea (he had no other wishes or concerns): he forbade extramarital sex. He was therefore a rather comical God, but let's not laugh at Alice for that. Of the Ten Commandments Moses gave to the people, fully nine didn't endanger her soul at all; she didn't feel like killing or not honoring her father, or coveting her neighbor's wife; only one commandment she felt to be not self-evident and therefore posed a genuine challenge: the famous seventh, which forbids fornication. In order to practice, show, and prove her religious faith, she had to devote her entire attention to this single commandment. And so out of a vague, diffuse, and abstract God, she created a God who was specific, comprehensible, and concrete: God Antifornicator*. (Kundera, 1980, p. 110)

Amidst the ongoing battle, Eduard undergoes a transformation of his reality as the lie he perpetuates becomes truth. Despite his initial project of pursuing worldly love, Eduard falls in love with Alice, and she becomes his primary focus:

*(...) these were weeks of torment. And the torment was that much greater because Eduard's desire for Alice was not only the desire of a body for a body; on the contrary, the more she refused him her body, the more lonesome and afflicted he became and the more he coveted her heart as well. However, neither her body nor her heart wanted to do anything about it; they were equally cold, equally wrapped up in themselves and self-satisfied*. (Kundera, 1980, p. 111)

The love that Eduard begins to feel for Alice has a discernible effect:

*(...) Alice's abrupt turnaround had occurred independently of his many weeks of persuasion, independently of his argumentation, independently of any logical consideration whatsoever. (...) because Alice herself kept chattering. She was cheerful, and nothing indicated that this turnaround in her soul had been dramatic or painful. When it got dark, they went back to the cottage, turned on the lights, turned down the bed, and kissed, whereupon Alice asked Eduard to turn off the lights. But the light of the stars continued to show through the window, so Eduard had to close the shutters as well at Alice's request. Then, in total darkness, Alice undressed and gave herself to him*. (Kundera, 1980, p. 123)

Everything had reached its culmination, and simultaneously, as Eduard's inclination and affection for Alice approached infinity, so did Alice's inclination and affection for Eduard. Alice, placing her trust in Eduard, assimilated his identity, embracing his pursuit of worldly love. Their intimate connection extended beyond a mere physical interaction, transcending the superficiality of two acrobats engaged in a carnal game. Likewise, Eduard, entrusting himself to Alice, adopted her identity, embracing her devotion to God and her faith. In his emotional realm, Eduard constructed a representation of Alice, engaging in a sacred sublimation where physical intimacy became unattainable. As they sought to find love within the preferred world of their respective partners, they found themselves engulfed in solitude:

*It was humiliating, terribly humiliating. The train idyllically clattered over the joints of the tracks (the girl was chattering), and Eduard said* —* Alice are you happy?*—* Yes, said Alice*. —* I'm miserable, said Eduard*. —* What, are you crazy? said Alice*. —* We shouldn't have done it. It shouldn't have happened*. —* What's gotten into you? You're the one who wanted to do it!*—* Yes, I wanted to, said Eduard. But that was my greatest mistake, for which God will never forgive me. It was a sin, Alice*. —* Come on, what's happened to you? asked the girl calmly*.—* You yourself always said that God wants love most of all!When Eduard heard Alice, after the fact, quietly appropriating the theological sophistries with which he had so unsuccessfully taken the field a while ago, fury seized him:* —* I said that to test you. Now I've found out how faithful you are to God! And a person who is capable of betraying God is capable of betraying a man a hundred times more easily!Alice always found ready answers, but it would have been better for her if she hadn't, because they only provoked his vindictive rage. Eduard went on and on talking (in the end he used the words “nausea” and “physical disgust”)—until he did obtain from this placid and gentle face (finally!) sobs, tears, and moans. Goodbye—he said to her at the station, and he left her in tears*. (Kundera, 1980, p. 187)

Eduard's most preferred project, the one with which he most identifies himself, is his *worldly love* project (a), the project of fornication, such that his identity *u*_1_(a) ∈ (1/2, 1] is so high that it is almost equal to 1; almost in the totality of his being, he is fornication. At the same time, the identity *u*_1_(b) ∈ [0, 1/2) of Eduard with the *God-loving* project (b), with which he least identifies, is almost equal to 0. Alice is the opposite of Eduard. However, in the difference between one and the other, they fall in love, and in game of the strategic interactions of ridiculous loves, Eduard in the project of *love to God* (b) looks for Alice, with a relative frequency (probability) equal to *u*_1_(a), almost equal to 1, at the same time that Alice in the project of *worldly love* (a) looks for Eduard, with a relative frequency (probability) *u*_2_(b), almost equal to 1. Almost with certainty, Eduard in the project of *love to God* (b) looks for Alice, and almost with certainty Alice in the project of *worldly love* (a) looks for Eduard, and in the Nash equilibrium in mixed strategies of the game of ridiculous loves, almost with certainty, the outcome is loneliness (see Figure 1). In Eduard's case, his most preferred project, the one that he identifies with the most, is his worldly love project (a)—the project of fornication. As a result, his identity *u*_1_(a) ∈ (1/2, 1] is almost equal to 1, meaning that he is almost entirely associated with fornication. Conversely, his identity *u*_1_(b) ∈ [0, 1/2) with the God-loving project (b), the project with which he least identifies, is almost equal to 0. Alice, on the other hand, is the opposite of Eduard. However, despite their differences, they fall in love with each other through strategic interactions in the game of ridiculous loves. Eduard looks for Alice in the project of love to God (b) with a relative frequency (probability) almost equal to 1, while Alice looks for Eduard in the project of worldly love (a) with a relative frequency (probability) almost equal to 1. In the Nash equilibrium of the game of ridiculous loves, the outcome is almost certainly loneliness (see Figure 1—*Of courtly love*).

In other words, Alice assimilates Eduard's project as her own, and follows Eduard in his worldly love project with the same intensity *u*_2_(b) as she follows her own God-loving project, creating a contradiction. With Julian Marias' concept of internalization, it can be said that in the strategic interaction of ridiculous love between Eduard and Alice, Alice has internalized Eduard and his ethic of fornication, while Eduard has internalized Alice and her faith in a non-fornicating God. This internalization creates a paradoxical situation where their beliefs and values are in conflict with their actions, resulting in a strategic interaction that defies rational analysis.

*In the case of falling in love in the strict sense, when people fall in love, they experience something that is a transformation; that is, the one who is in love is different from who he or she was before—this is clear. Their reality has been affected by this frequently sudden illusion of being in love. The fact that a person who, in principle, was a stranger—someone toward whom they might have had loving feelings or projected their desires—becomes an ingredient of their own reality. I mean by this that if a personal X-ray could be taken of someone, the other person, the one who is the object of their love, the one they are in love with, would be discovered in their own reality, in their interiority*. (Marías, 2015, p. 58)

In this possible world of courtly love, where lovers strive to find each other, the likelihood of them ending up alone is high. The strategic interaction of the game of ridiculous loves results in a state of loneliness for the players involved.

#### 3.1.1 The fictitious play process

Suppose now that *G* = [{*S*_1_, *S*_2_}, {*u*_1_, *u*_2_}] is a repeated game and consider a learning process associated with the structure of the game: the fictitious play process. For *t* = 1, 2, 3, ..., the sequence (p(*t*), q(*t*)) is a discrete fictitious play process if (p(*t*), q(*t*)) ∈ △(*S*_1_) × △(*S*_2_) such that (p(1), q(1)) is chosen by Nature and for each *t* = 2, 3, 4, ..., its hold that where b^*i*^(*t*−1) is the decision made by player *i* in period *t*, taking into consideration the information gathered up to stage *t* − 1.


(12) $$\begin{array}{l} \left(\text{p}\left(t\right),\text{q}\left(t\right)\right)=\left(\frac{\left(t-2\right)\cdot \text{p}\left(t-1\right)+{\text{b}}^{1}\left(t-1\right)}{t-1},\frac{\left(t-2\right)\cdot \text{q}\left(t-1\right)+{\text{b}}^{2}\left(t-1\right)}{t-1}\right) \end{array}$$

We affirm that for each time period *t*, *p*(*t*) is a prediction that player 2 makes regarding the probability of player 1 choosing action a at time *t*. Similarly, for each time period *t*, *q*(*t*) is a prediction that player 1 makes about the probability of player 2 choosing action a at time *t*. The forecast made by player *i*=1,2 for time period *t* is made after observing the history of event realizations up to the previous period ${\left({\text{b}}^{i}\left(t\right)\right)}_{s=1}^{t-1}$ (see Table 1.A). The best response functions of the players are


(13) $$BR_{1}\left(q\left(\text{t}\right)\right)=\{\begin{array}{cc} e_{1} & \left(q,1-q\right)\cdot M_{11:}>\left(q,1-q\right)\cdot M_{12:}\\ e_{2} & \text{otherwise} \end{array}$$

and


(14) $$BR_{2}\left(p\left(\text{t}\right)\right)=\{\begin{array}{cc} e_{1} & \left(p,1-p\right)\cdot M_{2:1}>\left(p,1-p\right)\cdot M_{2:2}\\ e_{2} & \text{otherwise} \end{array}$$

**Table 1.A:** Algorithm.

For each *i* = 1, 2:
Input
*u*_*i*_(a), *u*_*i*_(b), *k*_*i*_ and *M*_*i*_
*n*: number of rounds
initial conditions p(1),q(1) such that
$\text{p}={\left(\text{p}\left(t\right)\right)}_{t=1}^{n}$ and p[*t*] = p(*t*)
$\text{q}={\left(\text{q}\left(t\right)\right)}_{t=1}^{n}$ and q[*t*] = q(*t*)
Output
Step 1: construct the initial conditions of the vectors:
b^*i*^[*t*] = b^*i*^(*t*) such that ${\text{b}}^{i}={\left({\text{b}}^{i}\left(t\right)\right)}_{t=1}^{n}$
*v*_1_(a)[*t*] = *v*_1_(a, q(*t*)) such that $v_{1}\left(\text{a}\right)={\left(v_{1}\left(\text{a},\text{q}\left(t\right)\right)\right)}_{t=1}^{n}$
*v*_1_(b)[*t*] = *v*_1_(b, q(*t*)) such that $v_{1}\left(\text{b}\right)={\left(v_{1}\left(\text{b},\text{q}\left(t\right)\right)\right)}_{t=1}^{n}$
*v*_2_(a)[*t*] = *v*_2_(p(*t*), a) such that $v_{2}\left(\text{a}\right)={\left(v_{2}\left(\text{p}\left(t\right),\text{a}\right)\right)}_{t=1}^{n}$
*v*_2_(b)[*t*] = *v*_2_(p(*t*), b) such that $v_{2}\left(\text{b}\right)={\left(v_{2}\left(\text{p}\left(t\right),\text{b}\right)\right)}_{t=1}^{n}$
Step 2: initiate an loop:
for *t* in range (1, *n*):
p_1_[*t*]=sum(b^1^[*t*])/*t* such that p_1_[*t*] is the first component of p[*t*]
q_1_[*t*]=sum(b^2^[*t*])/*t* such that q_1_[*t*] is the first component of q[*t*]
*v*_1_(a)[*t*] = *M*_1_(1, 1)·q_1_[*t*]+*M*_1_(2, 1)·(1 − q_1_[*t*])
*v*_1_(b)[*t*] = *M*_1_(1, 2)·q_1_[*t*]+*M*_1_(2, 2)·(1 − q_1_[*t*])
*v*_2_(a)[*t*] = *M*_2_(1, 1)·p_1_[*t*]+*M*_2_(2, 1)·(1 − p_1_[*t*])
*v*_2_(b)[*t*] = *M*_2_(1, 2)·p_1_[*t*]+*M*_2_(2, 2)·(1 − p_1_[*t*])
if *v*_1_(a)[*t*]≥*v*_1_(b)[*t*]:
b^1^[*t*] = 1
else:
b^1^[*t*] = 0
if *v*_2_(a)[*t*]>*v*_2_(b)[*t*]:
b^2^[*t*] = 1
else:
b^2^[*t*] = 0


Therefore, the game *G* is played by *fictitious play* if, for each *t* its hold that


(15) $$\begin{array}{l} \left({\text{b}}^{1}\left(t\right),{\text{b}}^{2}\left(t\right)\right)=\left(BR_{1}\left(\text{q}\left(t\right)\right),BR_{2}\left(\text{p}\left(t\right)\right)\right) \end{array}$$

Fictitious play is a simple behavioral description of strategy choices for players in a repeated game. During fictitious play process, participants always remember relative frequency of each strategy that the opponent adopted. At each period, participants compute the expected payoff of all strategies taken by themselves according to the opponents' strategy distribution, and choose a strategy with the highest expected payoff. Memory, which comes from cognitive psychology, is considered to be inseparable with learning: Learning is a memory-based experience and rules-accumulation process. The algorithm (see Table 1.A) describes the implementation of the best response functions (13) and (14) within the simulation environment.

As the game *G* possesses the fictitious play property, there will be convergence toward one of the equilibria in pure strategies. In consequence, every limit point of every sequence (b^1^(*t*), b^2^(*t*)) generated by a fictitious play process is a Nash equilibrium of *G*. Hence, every such sequence converges to the closed set of Nash equilibria.

We consider the following parameterization for the Game of Courtly Love: Player 1, given their preferences, values projects a and b with weights of 0.9 and 0.1, respectively. On the other hand, Player 2, given their preferences, values projects a and b with weights of 0.1 and 0.9, respectively (see Table 1.B). The players have heterogeneous preferences; however, each one experiences empathy for the other, such that the empathy coefficient of both players is equal to 1.000.000. The simulation of the game allows us to study the interaction between two individuals who, despite having heterogeneous preferences, feel a high level of empathy toward each other. With the parameterization indicated in Table 1.B, it holds that for each (p(1), q(1)) such that *p*(1) ∈ (1/10, 1] and *q*(1) ∈ [0, 9/10) it holds that the sequence ${\left({\text{b}}^{1}\left(t\right),{\text{b}}^{2}\left(t\right)\right)}_{s=1}^{t-1}$ converges to ((1/10, 9/10), (9/10, 1/10)) (see Figure 1, Tables 1.A, 1.B).

**Table 1.B:** Parameters of the fictitious game process for the courtly love game.

**Player**	**Parameters**			**Equilibria nash**	
*i*	*u*_*i*_(**a**)	*u*_*i*_(**b**)	*k*_*i*_	σ_*i*_(a)	σ_*i*_(b)
1	0.9	0.1	1.000.000	0.1	0.9
2	0.1	0.9	1.000.000	0.9	0.1

The simulation of the Game of Courtly Love demonstrates that each individual selects the project most preferred by their counterpart, and in this simultaneous choice, the outcome is the mismatch, which is interpreted as an outcome of separation, death, or loss (see Table 1.C).

**Table 1.C:** Simulation of the game/fictitious play process.

***t***	**b^1^[*t*]**	**b^2^[*t*]**	**p_1_[*t*]**	**q_1_[*t*]**	***t***	**b^1^[*t*]**	**b^2^[*t*]**	**p_1_[*t*]**	**q_1_[*t*]**
1	0	1	0.8000	0.8000	1	0	1	0.8000	0.2000
2	1	0	0.0000	1.0000	2	1	0	0.0000	1.0000
3	0	1	0.5000	0.5000	3	0	1	0.5000	0.5000
4	0	1	0.3333	0.6666	4	0	1	0.3333	0.6666
5	0	1	0.2500	0.7500	5	0	1	0.2500	0.7500
⋯	⋯	⋯	⋯	⋯	⋯	⋯	⋯	⋯	⋯
996	0	1	0.1005	0.8994	996	0	1	0.1005	0.8994
997	0	1	0.1004	0.8995	997	0	1	0.1004	0.8995
998	0	1	0.1003	0.8996	998	0	1	0.1003	0.8996
999	0	1	0.1002	0.8998	999	0	1	0.1002	0.8998
1000	0	1	0.1001	0.8999	1000	0	1	0.1001	0.8999
1	0	1	0.2000	0.2000	1	0	1	0.5000	0.5000
2	1	0	0.0000	1.0000	2	1	0	0.0000	1.0000
3	0	1	0.5000	0.5000	3	0	1	0.5000	0.5000
4	0	1	0.3333	0.6666	4	0	1	0.3333	0.6666
5	0	1	0.2500	0.7500	5	0	1	0.2500	0.7500
⋯	⋯	⋯	⋯	⋯	⋯	⋯	⋯	⋯	⋯
996	0	1	0.1005	0.8994	996	0	1	0.1005	0.8994
997	0	1	0.1004	0.8995	997	0	1	0.1004	0.8995
998	0	1	0.1003	0.8996	998	0	1	0.1003	0.8996
999	0	1	0.1002	0.8998	999	0	1	0.1002	0.8998
1,000	0	1	0.1001	0.8999	1,000	0	1	0.1001	0.8999

The red values indicate the initial conditions (starting points) of the simulation process. These values correspond to the initial probabilities assigned at *t* = 1 before the iterative fictitious play updates.

The institutionalization of the code of conduct and behavioral pattern represented by Courtly Love defines an ideology of “romantic love” that has exhibited historical persistence (Judd, 1985).

Romantic love frequently embodies the notion of a tragic ending in Western culture, being widely portrayed within the cinematic realm across numerous films. These films depict narratives where lovers encounter insurmountable obstacles, emotional challenges, and societal barriers, culminating in a tragic outcome such as separation, death, or loss. Romantic love as passionate affection, with all its excesses, and furthermore, not being solely confined to cinematic fiction, entails a form of violence in adolescent romantic relationships: Verbal abuse, physical and sexual abuse, threats, rape, and murder.

Among young individuals, the idealization of love and romantic myths are highly prevalent due to our culture and society, which subsequently lead them to foster dysfunctional relationships that, in some manner, promote and facilitate intimate partner violence and outcomes of discordance (Martín-Salvador et al., 2021; Pascual-Fernández, 2016). Therefore, we have reached the following corollary.

Corollary 3. *Of courtly love* —. If Melibea has become Calisto's project and therefore his highest priority *k*_1_ → ∞, while at the same time Calisto has become Melibea's project and therefore her highest priority *k*_2_ → ∞, it follows that


(16) $${\left(p^{\text{*}},q^{\text{*}}\right)}^{⊤}=\left(\left(u_{1}\left(b\right),u_{1}\left(a\right)\right),\left(u_{2}\left(b\right),u_{2}\left(a\right)\right)\right)$$

is the NEMS of the game of courtly love. In the fictitious play process, it holds that for every (p(1), q(1)) such that *p*(1) ∈ (*u*_1_(b), 1] and *q*(1) ∈ [0, *u*_2_(b)) hold true,


(17) $$\begin{array}{l} \underset{t\to \infty }{\text{lim}}{\left({\text{b}}^{1}\left(s\right),{\text{b}}^{2}\left(s\right)\right)}_{s=1}^{t}=\left(\left(u_{1}\left(\text{b}\right),u_{1}\left(\text{a}\right)\right),\left(u_{2}\left(\text{b}\right),u_{2}\left(\text{a}\right)\right)\right) \end{array}$$

is satisfied.

#### 3.1.2 Social choice and power structures in courtly love

Courtly love, as a manifestation of specific cultural norms, can be viewed as a form of social choice that reflects and reinforces the cultural and social structures of its time, while also shaping individual preferences within a collective context. The proposed theoretical model of courtly love, along with the implemented simulation, elucidates the phenomenon of disillusionment or unrequited love based on the mutual idealization of lovers—an aspect distinctive of courtly love culture. Mutual idealizations create emotional and physical distances that often lead to disenchantment and unrequited love. This mutual idealization involves a complex power structure: while the lover is subordinate to the lady, which can lead to a unidimensional experience of love, the woman is also subordinated to the man in a patriarchal relationship, being perceived as an object of desire rather than an autonomous agent with her own agency. The mutual idealization embodies a power structure that gives rise to social norms as an expression of the social choices of the era. Courtly love reflects how romantic decisions and behaviors are influenced by social and cultural expectations surrounding idealization—both the idealization of the woman by the man within the patriarchal framework and the idealization of the man by the woman in her quest for a patriarchal provider.

### 3.2 *Of hedonist love*

The concept of happiness in contemporary Western society is primarily founded on a hedonistic criterion. In the present narrative, Eduard and Alice regulate their lives based on the principle of pleasure. For Eduard and Alice, their ultimate goal is to achieve happiness through the pursuit of pleasure, to have fun:

*(...) Having fun lies in the satisfaction of consuming and “taking in” commodities, sights, food, drinks, cigarettes, people, lectures, books, movies—all are consumed, swallowed. The world is one great object for our appetite, a big apple, a big bottle, a big breast; we are the sucklers, the eternally expectant ones, the hopeful ones—and the eternally disappointed ones. Our character is geared to exchange and to receive, to barter and to consume; everything, spiritual as well as material objects, becomes an object of exchange and of consumption*. (Fromm, 1956, p. 67)

In modern Western culture, “love” has become just another product that can be purchased or acquired through various attributes. In this story, Eduard's focus is on constructing or acquiring a specific profile of social, economic, and physical attributes in order to be desired and chosen by Alice. Rather than developing empathy for Alice and getting to know her as a human being with desires and contradictions, Eduard is preoccupied with achieving a certain status or appearance that will make him attractive to her:

*Our whole culture is based on the appetite for buying, on the idea of a mutually favorable exchange. Modern man's happiness consists in the thrill of looking at the shop windows, and in buying all that he can afford to buy, either for cash or on installments. He (or she) looks at people in a similar way. For the man an attractive girl—and for the woman an attractive man—are the prizes they are after. “Attractive” usually means a nice package of qualities which are popular and sought after on the personality market*. (Fromm, 1956, p. 67)

In contemporary society, a market of men and women exists where every individual, both men and women, strive to be chosen by their desired partner. The goal is to engage in casual sexual encounters without commitment, with the possibility of transitioning from a project of fornication to one of intimacy in the affections and the communion of their private worlds (Carpenter and McEwan, 2016). This metaphorical market of love and desire, once described by (Fromm 1956) as a concept, has materialized through various online dating platforms such as Tinder:

*The Tinder app was launched in 2012 and is today the most popular of the numerous online dating platforms. Tinder was privately founded and was purchased by the Match Group in 2017 for 1 billion US bollars. The newest estimate of the worth of Tinder just hit 10 billion US dollars. Tinder counts about 57 million users of which 10 million are active on a daily basis in 196 countries around the world. 80% of Tinder users are between 18 and 34 years old, and 62% are male, 38% female. 54% are singles, 12% are in a relationship and 30% are married and even though the app's origin is North American, only 40% of the users today reside there (Brandt*, *2019**). Tinder has been by now translated into 40 languages and generates 26 million matches per day. These lead to 1 million dates a week with a serious implication for the practice of relationships; in the USA, up to 16% (in 2017) of married or engaged couples actually met on Tinder (ibd.). There are other striking significant dynamics showing the impact of Tinder in everyday life, such as Tinder themed weddings (Lopez et al.*, *2019**) and merchandise for so-called Tinderbabies (Kravitz*, *2018**)*. (Degen and Kleeberg-Niepage, 2022, p. 182)

In the market of personality on Tinder, the equilibrium strategy is that of short-selling. Initially, Eduard's profile is priced at a high value on Tinder, where it serves as his display counter. Selling his profile at a high price increases his chances of being chosen by a large number of Alice, from whom he can choose. However, as Eduard chooses the profiles he prefers and matches with, his price drops as Alice gets to know him beyond his photo profile. This may lead to an inevitable disappointment with a high probability:


$$BR_{1}\left(q\right)=\{\begin{array}{l} \begin{array}{ccccc} \left\{p^{\text{*}}:\text{p=0}\right\} & \text{if} & q<u_{2}\left(b\right)-u_{1}/k_{1} & \text{such that} & \left(u_{2}\left(b\right)-u_{1}/k_{1}\right)\to 0\\ \left\{p^{\text{*}}:\text{0}\leq \text{p}\leq \text{1}\right\} & \text{if} & q=u_{2}\left(b\right)-u_{1}/k_{1} & \text{such that} & \left(u_{2}\left(b\right)-u_{1}/k_{1}\right)\to 0\\ \left\{p^{\text{*}}:\text{p=1}\right\} & \text{if} & q>u_{2}\left(b\right)-u_{1}/k_{1} & \text{such that} & \left(u_{2}\left(b\right)-u_{1}/k_{1}\right)\to 0 \end{array} \end{array}$$

*(...) she visited Los Angeles in the summer of 2017, met a guy through the app, hung out with him twice, and then stayed in touch by phone. They bonded over their childhoods and “leftist ideologies.” Soon, she had moved from Ohio to live with him in California, but quickly found his apartment too messy, his “affinity for drinking” too gross and his “large hair-shedding dog” too destructive. As for their shared ideology? In the end, she wrote, he turned out to be “a total brocialist.”* (Bromwich, 2018)

In the context of the Tinder personality market, the optimal strategy for Eduard is to engage in short-selling. Initially, Eduard's profile on Tinder is priced high due to his carefully selected photo profile, which aims to maximize his “beauty” and increase his chances of being chosen by a larger number of women. However, as Eduard selects and matches with his preferred profiles, the value of his profile will decrease at a later stage since each Alice he matches with will know him beyond his photo profile, and there is a high probability of disappointment in the interaction, given that Eduard's ultimate goal is a hedonistic cult of the self, where emotional investment is of low priority. At the end of the encounters, Eduard will remain in the women's market with his active profile on Tinder, but with the women he matched with, the price of his profile will be low, such that his benefit will have been the physical pleasure obtained. Eduard's level of empathy toward Alice is almost non-existent, causing his parameter *k*_1_ in the game of ridiculous loves to converge toward *u*_1_/*u*_2_(b). The same holds true for Alice, and her parameter *k*_2_ converges toward *u*_2_/*u*_1_(a). Eduard and Alice can be classified as sociosexual individuals, who exhibit a consistent pattern of seeking out casual sexual encounters with multiple partners while displaying low levels of empathy and high levels of narcissism (Botnen et al., 2018). Formally, Eduard and Alices are sociosexual if, and only if,

**1**. *k*_1_→*u*_1_/*u*_2_(b) and (*u*_1_(a), *u*_1_(b)) → (1, 0)

**2**. *k*_2_→*u*_2_/*u*_1_(a) and (*u*_2_(a), *u*_2_(b)) → (0, 1)

**Figure 2:**
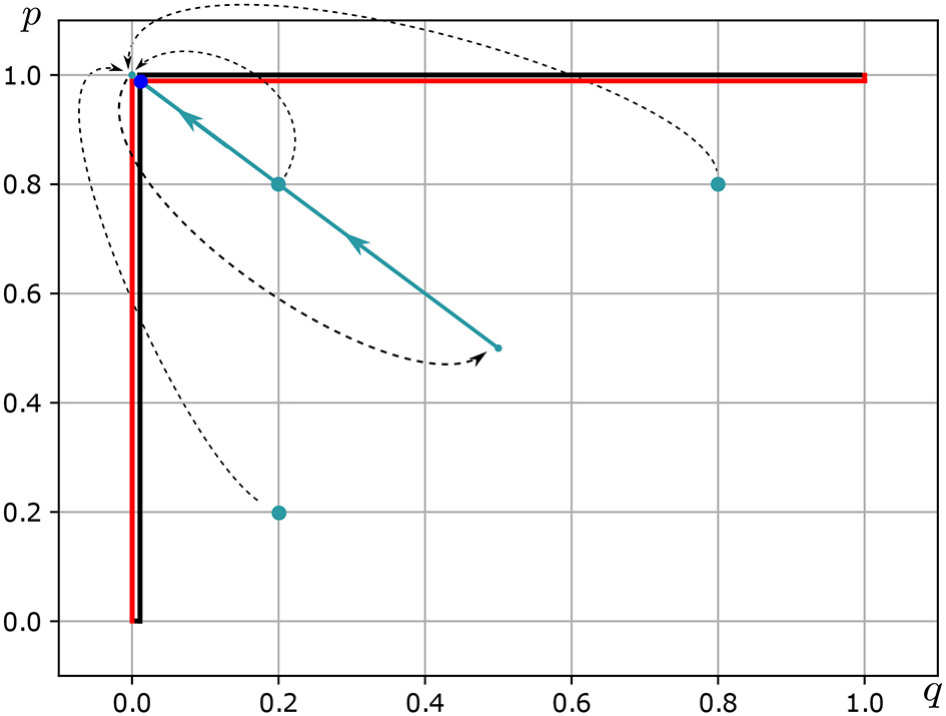
Nash equilibrium in mixed strategies *Of Hedonist Love*. Source: Own elaboration.

In the Tinder market, with a high probability, each match will result in loneliness. The Eduard's best response correspondence *BR*_1_:△(*S*_2_)⇉△(*S*_1_) is as follows (see Figure 2—Red Line):

The Alice's best response correspondence *BR*_2_:△(*S*_1_)⇉△(*S*_2_) is as follows (see Figure 2—Black Line):


$$BR_{2}\left(p\right)=\{\begin{array}{l} \begin{array}{ccccc} \left\{q^{\text{*}}:\text{q=0}\right\} & \text{if} & p<u_{1}\left(b\right)+u_{2}/k_{2} & \text{such that} & \left(u_{1}\left(b\right)+u_{2}/k_{2}\right)\to 1\\ \left\{q^{\text{*}}:\text{0}\leq \text{q}\leq \text{1}\right\} & \text{if} & p=u_{1}\left(b\right)+u_{2}/k_{2} & \text{such that} & \left(u_{1}\left(b\right)+u_{2}/k_{2}\right)\to 1\\ \left\{q^{\text{*}}:\text{q=1}\right\} & \text{if} & p>u_{1}\left(b\right)+u_{2}/k_{2} & \text{such that} & \left(u_{1}\left(b\right)+u_{2}/k_{2}\right)\to 1 \end{array} \end{array}$$

In consequence, the NEMS of the game *of hedonist love* is


$$\begin{array}{l} \left({\text{p}}^{*},{\text{q}}^{*}\right)=\left(\left(u_{1}\left(\text{b}\right)+u_{2}/k_{2},u_{1}\left(\text{a}\right)-u_{2}/k_{2}\right),\left(u_{2}\left(\text{b}\right)-u_{1}/k_{1},u_{2}\left(\text{a}\right)+u_{1}/k_{1}\right)\right) \end{array}$$

In this scenario, given the low levels of empathy within the context of a hedonistic conception of love, the model predicts that Edward will choose his most preferred project (**a**) with almost certainty, while Alice will choose her most preferred project (**b**) with the same level of certainty.

Consequently, the rational choice model enables us to forecast that low levels of empathy between two individuals lead to an outcome that is almost certainly one of discord and the dissolution of the romantic relationship. Precisely, within the realm of psychology, (Jonason et al. 2012) and (Tsoukas and March 2018) find that the likelihood of short-term relationships is notably high when there are diminished levels of commitment. In the scenario where Edward and Alice perceive their potential partners as “consumable objects” within the framework of a hedonistic conception of love, each individual necessitates their partner to conform to a specific set of characteristics that align with their idealized model of a partner. Consequently, when Edward selects the “like” option on Alice's Tinder profile, it is because Alice possesses these qualities, which further ignite Edward's desire to “consume” her, extending beyond a purely sexual context. In this plausible reality, both Edward and Alice pursue love, yet ultimately encounter solitude, ensnared in the absurd game of hedonistic love. The theoretical model proposed within the framework of game theory enables us to underpin a highly specific logic of behavior concerning love, which aligns with the explanatory mechanisms presented in the theory. The added value of our theory lies in demonstrating that the confluence of institutions, information, and objectives allows for the explicit elucidation of the principle of rationality underpinning this hedonistic behavioral logic regarding love.

#### 3.2.1 The fictitious play process

Suppose now that *G* = [{*S*_1_, *S*_2_}, {*u*_1_, *u*_2_}] is a repeated game. Applying the algorithm described in Table 1.A, which simulates the learning process associated with the structure of the Game of Hedonist Love, we consider the following initial conditions: Player 1, given their preferences, values projects a and b with weights of 0.9 and 0.1, respectively.

On the other hand, Player 2, given their preferences, values projects a and b with weights of 0.1 and 0.9, respectively. Both players have an empathy coefficient equal to 0.9 (see Table 2.A). In general, for any (p(1), q(1)) such that *p*(1) ∈ [0, 99/100) and *q*(1) ∈ (1/100, 1] it holds that for *t*≥126, the sequence (b^1^(*t*), b^2^(*t*)) converges to (see Figure 2 and Table 2.B).


(18) $$\begin{array}{l} \left(\left(u_{1}\left(\text{b}\right)+\frac{u_{2}}{k_{2}},u_{1}\left(\text{a}\right)-\frac{u_{2}}{k_{2}}\right),\left(u_{2}\left(\text{b}\right)-\frac{u_{1}}{k_{1}},u_{2}\left(\text{a}\right)+\frac{u_{1}}{k_{1}}\right)\right) \end{array}$$

**Table 2.A:** Parameters of the fictitious game process for the hedonist love game.

**Game**	**Player**	**Parameters**			**Equilibria Nash**	
	*i*	*u*_*i*_(**a**)	*u*_*i*_(**b**)	*k*_*i*_	σ_*i*_(a)	σ_*i*_(b)
*Of Hedonist Love*	1	0.9	0.1	0.9	0.1	0.9
	2	0.1	0.9	0.9	0.9	0.1

**Table 2.B:** Simulation of the game/fictitious play process.

***t***	**b^1^[*t*]**	**b^2^[*t*]**	**p_1_[*t*]**	**q_1_[*t*]**	***t***	**b^1^[*t*]**	**b^2^[*t*]**	**p_1_[*t*]**	**q_1_[*t*]**
1	1	0	0.8000	0.8000	1	1	0	0.8000	0.2000
2	0	1	1.0000	0.0000	2	0	1	1.0000	0.0000
3	1	0	0.5000	0.5000	3	1	0	0.5000	0.5000
4	1	0	0.6666	0.3333	4	1	0	0.6666	0.3333
5	1	0	0.7500	0.2500	5	1	0	0.7500	0.2500
⋯	⋯	⋯	⋯	⋯	⋯	⋯	⋯	⋯	⋯
996	1	0	0.9879	0.0120	996	1	0	0.9879	0.0120
997	1	0	0.9879	0.0120	997	1	0	0.9879	0.0120
998	1	0	0.9879	0.0120	998	1	0	0.9879	0.0120
999	1	0	0.9879	0.0120	999	1	0	0.9879	0.0120
1,000	1	0	0.9879	0.0120	1,000	1	0	0.9879	0.0120
1	1	0	0.2000	0.2000	1	1	0	0.5000	0.5000
2	0	1	1.0000	0.0000	2	0	1	1.0000	0.0000
3	1	0	0.5000	0.5000	3	1	0	0.5000	0.5000
4	1	0	0.6666	0.3333	4	1	0	0.6666	0.3333
5	1	0	0.7500	0.2500	5	1	0	0.7500	0.2500
⋯	⋯	⋯	⋯	⋯	⋯	⋯	⋯	⋯	⋯
996	1	0	0.9879	0.0120	996	1	0	0.9879	0.0120
997	1	0	0.9879	0.0120	997	1	0	0.9879	0.0120
998	1	0	0.9879	0.0120	998	1	0	0.9879	0.0120
999	1	0	0.9879	0.0120	999	1	0	0.9879	0.0120
1,000	1	0	0.9879	0.0120	1,000	1	0	0.9879	0.0120

The red values indicate the initial conditions (starting points) of the simulation process. These values correspond to the initial probabilities assigned at *t* = 1 before the iterative fictitious play updates.

Therefore, we have reached the following corollary.

Corollary 4. *Of Hedonist Love*—. If Eduard and Alice are sociosexual persons then


(19) $$\begin{array}{l} {\left(\text{p*},\text{q*}\right)}^{⊤}=\left(\left(u_{1}\left(\text{b}\right)+\frac{u_{2}}{k_{2}},u_{1}\left(\text{a}\right)-\frac{u_{2}}{k_{2}}\right),\left(u_{2}\left(\text{b}\right)-\frac{u_{1}}{k_{1}},u_{2}\left(\text{a}\right)+\frac{u_{1}}{k_{1}}\right)\right) \end{array}$$

is the NEMS of the game *of hedonist love*. In the fictitious play process, it holds that for every (p(1), q(1)) such that $p\left(1\right)\in \left(0,u_{1}\left(\text{b}\right)+\frac{u_{2}}{k_{2}}\right]$ and $q\left(1\right)\in \left(u_{2}\left(\text{b}\right)-\frac{u_{1}}{k_{1}},1\right]$ hold true, is satisfied.


(20) $$\begin{array}{l} \underset{t\to \infty }{\text{lim}}{\left({\text{b}}^{1}\left(s\right),{\text{b}}^{2}\left(s\right)\right)}_{s=1}^{t}=\left(\left(u_{1}\left(\text{b}\right)+\frac{u_{2}}{k_{2}},u_{1}\left(\text{a}\right)-\frac{u_{2}}{k_{2}}\right),\left(u_{2}\left(\text{b}\right)-\frac{u_{1}}{k_{1}},u_{2}\left(\text{a}\right)+\frac{u_{1}}{k_{1}}\right)\right) \end{array}$$

#### 3.2.2 Social choice and power structures in hedonist love

In hedonistic love, individuals present themselves as “products” seeking “consumers,” implying a transactional approach where people aim to maximize pleasure and personal satisfaction in a context of very low empathy. The hedonistic love model elucidates the impact of a power structure based on appearance, physical attractiveness, and immediate desire on pairing decisions, resulting in disillusionment and estrangement. This power structure places crucial emphasis on superficial valuation and aesthetics. Social choice is manifested in the social norms surrounding matching rules, which strongly shape individual preferences based on the “objectification” of partners in a low-empathy context. It is evident that hedonistic love champions individual autonomy and freedom within a cultural context of low empathy, characterized by the pursuit of instant gratification and a lack of long-term commitment. The eventual outcome is disillusionment and estrangement. The proposed hedonistic love model captures this dynamic clearly. This power structure underscores a tendency toward ephemeral relationships and a reduced willingness to invest in the development of lasting and meaningful connections.

### 3.3 *Of patriarchal love*

**Figure 3:**
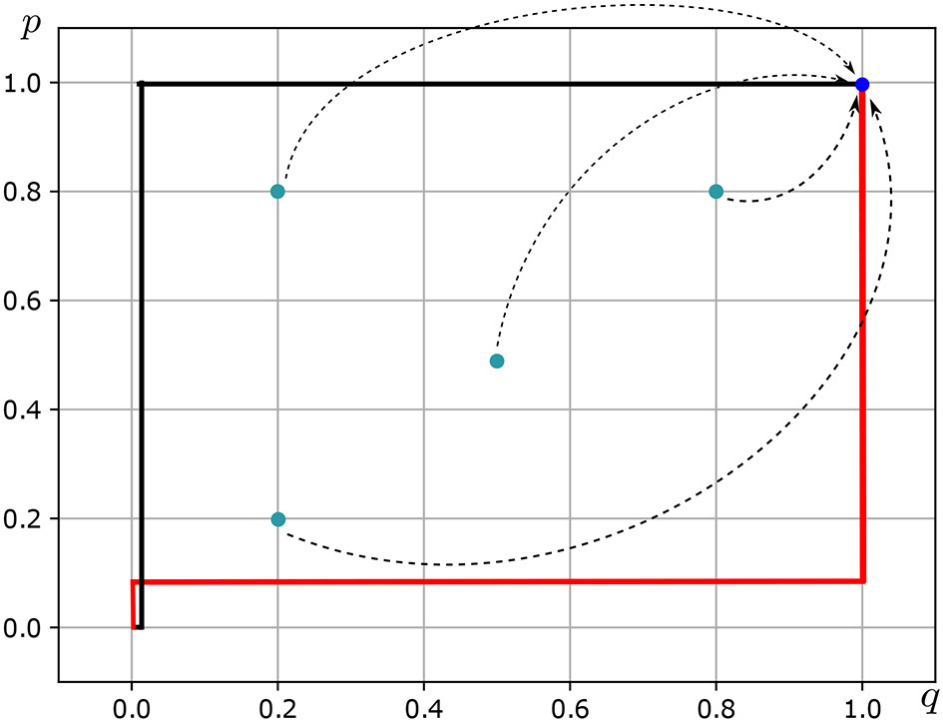
Nash equilibrium in mixed strategies *Of Patriarchal Love*. Source: Own elaboration.

In a patriarchal society, the family holds the utmost significance as an institution. In this realm, the archetypal woman of patriarchy is a sweet and loving figure, devoted to the family project (b) with all her life, desires, and aspirations. Consequently, b becomes the preferred project for the woman of patriarchy, leading to *u*_2_(b) converging to 1. The parameter *k*_2_, which represents the level of empathy, also converges to infinity, as the woman of patriarchy, accepting her sacred destiny in the family and bound by the obligation of obedience, is required to exhibit an infinite level of empathy. In contrast, the patriarchal man's objective is to continue the family line and, in the upper classes, to establish political and social alliances. In some extreme patriarchal societies, such as ancient Greek and Roman cultures, loving one's wife was deemed irrelevant (see Ovid's “Metamorphoses,” Aristotle's “Politics,” and Plato's “Republic.”). In consequence, for the patriarchal male, the parameter *k*_1_ converges to *u*_1_/*u*_2_(b). Politics, power, and wealth are the most desirable projects for the man of patriarchy, with *u*_1_(a) converging to 1. The patriarchal man's best response correspondence *BR*_1_:△(*S*_2_)⇉△(*S*_1_) is as follows (see Figure 3—Red line):


$$BR_{1}\left(q\right)=\{\begin{array}{l} \begin{array}{ccccc} \left\{p^{\text{*}}:\text{p=0}\right\} & \text{if} & q<u_{2}\left(b\right)-u_{1}/k_{1} & \text{such that} & u_{2}\left(b\right)-u_{1}/k_{1}\to 0\\ \left\{p^{\text{*}}:\text{0}\leq \text{p}\leq \text{1}\right\} & \text{if} & q=u_{2}\left(b\right)-u_{1}/k_{1} & \text{such that} & u_{2}\left(b\right)-u_{1}/k_{1}\to 0\\ \left\{p^{\text{*}}:\text{p=1}\right\} & \text{if} & q>u_{2}\left(b\right)-u_{1}/k_{1} & \text{such that} & u_{2}\left(b\right)-u_{1}/k_{1}\to 0 \end{array} \end{array}$$

The patriarchal woman's best response correspondence *BR*_2_:△(*S*_1_)⇉△(*S*_2_) is as follows (see Figure 3—Black line):


$$BR_{2}\left(p\right)=\{\begin{array}{l} \begin{array}{ccccc} \left\{q^{\text{*}}:\text{q=0}\right\} & \text{if} & p<u_{1}\left(b\right)+u_{2}/k_{2} & \text{such that} & u_{1}\left(b\right)+u_{2}/k_{2}\to u_{1}\left(b\right)\\ \left\{q^{\text{*}}:\text{0}\leq \text{q}\leq \text{1}\right\} & \text{if} & p=u_{1}\left(b\right)+u_{2}/k_{2} & \text{such that} & u_{1}\left(b\right)+u_{2}/k_{2}\to u_{1}\left(b\right)\\ \left\{q^{\text{*}}:\text{q=1}\right\} & \text{if} & p>u_{1}\left(b\right)+u_{2}/k_{2} & \text{such that} & u_{1}\left(b\right)+u_{2}/k_{2}\to u_{1}\left(b\right) \end{array} \end{array}$$

In consequence,


(21) $$\begin{array}{l} {\left({\text{p}}^{*},{\text{q}}^{*}\right)}^{⊤}=\left(\left(u_{1}\left(\text{b}\right)+u_{2}/k_{2},u_{1}\left(\text{a}\right)-u_{2}/k_{2}\right),\left(u_{2}\left(\text{b}\right)-u_{1}/k_{1},u_{2}\left(\text{a}\right)+u_{1}/k_{1}\right)\right) \end{array}$$

is the NEMS of the game *of patriarchal love* (see Figure 3).

#### 3.3.1 The fictitious play process

We suppose now that *G* = [{*S*_1_, *S*_2_}, {*u*_1_, *u*_2_}] is a repeated game. Applying the algorithm described in Table 1.A, which simulates the learning process associated with the structure of the Game of Patriarchal Love, we consider the following initial conditions: Player 1, given their preferences, values projects a and b with weights of 0.9 and 0.1, respectively. On the other hand, Player 2, given their preferences, values projects a and b with weights of 0.1 and 0.9, respectively. However, the empathy coefficients vary, with the coefficient for women being equal to 1.000.000 and for men equal to 0.9 (see Table 3.A).

**Table 3.A:** Parameters of the fictitious game process for the patriarchal love game.

**Game**	**Player**	**Parameters**			**Equilibria nash**	
	*i*	*u*_*i*_(**a**)	*u*_*i*_(**b**)	*k*_*i*_	σ_*i*_(a)	σ_*i*_(b)
*Of Patriarchal Love*	1	0.9	0.1	0.9	0.1	0.9
	2	0.1	0.9	1.000.000	0.01	0.98

In general, for any (p(1), q(1)) such that *p*(1) ∈ (1/10, 1] and *q*(1) ∈ (1/100, 1] it holds that for *t*≥126, the sequence ${\left({\text{b}}^{1}\left(t\right),{\text{b}}^{2}\left(t\right)\right)}_{s=1}^{t-1}$ converges to ((1, 0), (1, 0)) (see Figure 3, Tables 3.A, 3.B). In the game of patriarchal love, given the NEMS of the Patriarchal Love game, the probability is very close to 1 that the man pursues his objective of continuing his patriarchal lineage. Simultaneously, the patriarchal woman accompanies the patriarchal man in his most preferred project with a probability close to 1 (given her infinite love for him), dedicating her entire life, desires, and aspirations to the family project. Patriarchal love is highly stable, resulting in an unequal and submissive position for the woman. The NMES of patriarchy is a contract in which the patriarchal man and the patriarchal woman ensure the stability of the family; both are engaged in this common project, where the woman pays a very high price: submission and abuse.

**Table 3.B:** Simulation of the game/fictitious play process.

***t***	**b^1^[*t*]**	**b^2^[*t*]**	**p_1_[*t*]**	**q_1_[*t*]**	***t***	**b^1^[*t*]**	**b^2^[*t*]**	**p_1_[*t*]**	**q_1_[*t*]**
1	1	1	0.8000	0.8000	1	1	0	0.8000	0.2000
2	1	1	1.0000	1.0000	2	1	1	1.0000	1.0000
3	1	1	1.0000	1.0000	3	1	1	1.0000	1.0000
4	1	1	1.0000	1.0000	4	1	1	1.0000	1.0000
5	1	1	1.0000	1.0000	5	1	1	1.0000	1.0000
⋯	⋯	⋯	⋯	⋯	⋯	⋯	⋯	⋯	⋯
996	1	1	1.0000	1.0000	996	1	1	1.0000	1.0000
997	1	1	1.0000	1.0000	997	1	1	1.0000	1.0000
998	1	1	1.0000	1.0000	998	1	1	1.0000	1.0000
999	1	1	1.0000	1.0000	999	1	1	1.0000	1.0000
1,000	1	1	1.0000	1.0000	1,000	1	1	1.0000	1.0000
1	1	1	0.2000	0.2000	1	1	1	0.5000	0.5000
2	1	1	1.0000	1.0000	2	1	1	1.0000	1.0000
3	1	1	1.0000	1.0000	3	1	1	1.0000	1.0000
4	1	1	1.0000	1.0000	4	1	1	1.0000	1.0000
5	1	1	1.0000	1.0000	5	1	1	1.0000	1.0000
⋯	⋯	⋯	⋯	⋯	⋯	⋯	⋯	⋯	⋯
996	1	1	1.0000	1.0000	996	1	1	1.0000	1.0000
997	1	1	1.0000	1.0000	997	1	1	1.0000	1.0000
998	1	1	1.0000	1.0000	998	1	1	1.0000	1.0000
999	1	1	1.0000	1.0000	999	1	1	1.0000	1.0000
1,000	1	1	1.0000	1.0000	1,000	1	1	1.0000	1.0000

The red values indicate the initial conditions (starting points) of the simulation process. These values correspond to the initial probabilities assigned at *t* = 1 before the iterative fictitious play updates.

Therefore, we have reached the following corollary.

Corollary 5. *Of Patriarchal Love*—. Let *G* = [{*S*_1_, *S*_2_}, {***u***_1_, ***u***_2_}] be the game of love such that Eduard is the patriarch's man (*k*_1_→*u*_1_/*u*_2_(b) and *u*_1_(a) → 1) and Alice is the patriarch's woman (*k*_2_ → ∞ y *u*_2_(b) → 1). Therefore, it holds that


(22) $$\begin{array}{l} \left(\text{p*},\text{q*}\right)=\left(\left(u_{1}\left(\text{b}\right)+\frac{u_{2}}{k_{2}},u_{1}\left(\text{a}\right)-\frac{u_{2}}{k_{2}}\right),\left(u_{2}\left(\text{b}\right)-\frac{u_{1}}{k_{1}},u_{2}\left(\text{a}\right)+\frac{u_{1}}{k_{1}}\right)\right) \end{array}$$

is the NEMS of the game *of Patriarchal Love. In the fictitious play process, it holds that for every* (p(1), q(1)) *such that* $p\left(1\right)\in \left(u_{1}\left(\text{b}\right)+\frac{u_{2}}{k_{2}},1\right]$ *and* $q\left(1\right)\in \left(u_{2}\left(\text{b}\right)-\frac{u_{1}}{k_{1}},1\right]$ *hold true*,


(23) $$\begin{array}{l} \underset{t\to \infty }{\text{lim}}{\left({\text{b}}^{1}\left(s\right),{\text{b}}^{2}\left(s\right)\right)}_{s=1}^{t}=\left(\left(1,0\right),\left(1,0\right)\right) \end{array}$$

*is satisfied*.

#### 3.3.2 Social choice and power structures in patriarchal love

The proposed patriarchal love model describes the dominant position of the man, manifested in the low levels of empathy he experiences toward the woman, and the woman's subordination, manifested in her unconditional devotion to the man with a very high coefficient of empathy for him. This model allows us to recreate an environment where the man acts as the protector, provider, and head of the household, while the woman is obedient, submissive, and focused on domestic and caregiving roles. We assert that the patriarchal love model reflects how individual preferences are influenced by the patriarchal society. The internalization of these norms leads individuals to accept and perpetuate patriarchal structures of domination and unequal power reflected in their preferences. In this power structure, which reproduces gender inequality and limits women's autonomy and agency, ensuring male domination and control in relationships, we have demonstrated that the resulting equilibrium is stable pairing.

### 3.4 *Of well-balanced love*

**Figure 4:**
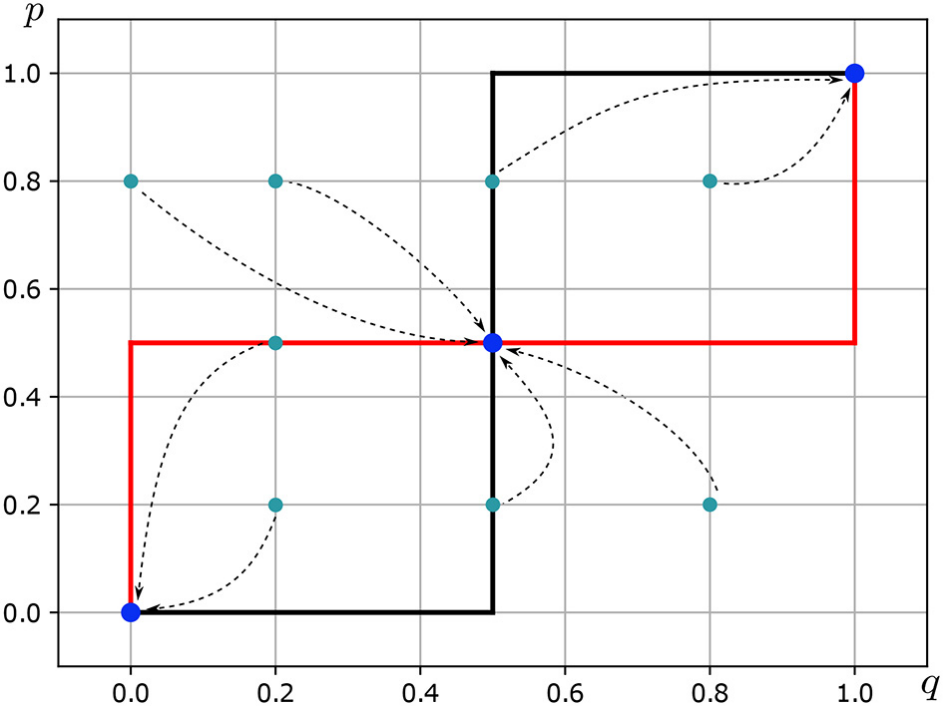
Nash equilibrium in mixed strategies of well-balanced love. Source: Own elaboration.

Let us consider a possible world in which Eduard is neither the man of patriarchalism, nor the fornicating man of Tinder, nor the man of l'amour courtois. Instead, let us envision a scenario where Eduard and Alice recognize each other as equals in rights and capabilities. In this world, Eduard and Alice are peers—professionally, politically, socially, and sexually. In this possible world, Eduard and Alice share the same preference structure such that *u*_1_(a) = *u*_2_(a) = 1/2 and *u*_1_(b) = *u*_2_(b) = 1/2. In consequence, it holds that *u*_1_ = *u*_1_(a)−*u*_1_(b) = 0 and *u*_2_ = *u*_2_(b)−*u*_2_(a) = 0. In this possible world, there is no place for excesses, whether narcissistic or of overflowing empathy that degenerates into alienation. Eduard's best response correspondence *BR*_1_:△(*S*_2_)⇉△(*S*_1_) is as follows (see Figure 4—Red Line):


$$BR_{1}\left(q\right)=\{\begin{array}{l} \begin{array}{ccc} \left\{p^{\text{*}}:\text{p=0}\right\} & \text{if} & q<1/2\\ \left\{p^{\text{*}}:\text{0}\leq \text{p}\leq \text{1}\right\} & \text{if} & q=1/2\\ \left\{p^{\text{*}}:\text{p=1}\right\} & \text{if} & q>1/2 \end{array} \end{array}$$

The Alice's best response correspondence *BR*_2_:△(*S*_1_)⇉△(*S*_2_) is as follows (see Figure 4—Black Line):


$$BR_{2}\left(p\right)=\{\begin{array}{l} \begin{array}{ccc} \left\{q^{\text{*}}:\text{q=0}\right\} & \text{if} & p<1/2\\ \left\{q^{\text{*}}:\text{0}\leq \text{q}\leq \text{1}\right\} & \text{if} & p=1/2\\ \left\{q^{\text{*}}:\text{q=1}\right\} & \text{if} & p>1/2 \end{array} \end{array}$$

In consequence, the NEMS of the game *of well-balanced love* is (p^*^, q^*^) = ((1/2, 1/2), (1/2, 1/2)).

#### 3.4.1 The fictitious play process

Let us assume that *G* = [{*S*_1_, *S*_2_}, {*u*_1_, *u*_2_}] is a repeated game such that we consider the following parameterization for the Game of well-balanced love: Player 1, given their preferences, values projects a and b with weights of 0.5 and 0.5, respectively. On the other hand, Player 2, given their preferences, values projects a and b with weights of 0.5 and 0.5, respectively. The empathy coefficient of each player is equal to 10 (see Table 4.A).

**Table 4.A:** Parameters of the fictitious game process for the well-balanced love.

**Game**	**Player**	**Parameters**			**Equilibria Nash**	
	*i*	*u*_*i*_(**a**)	*u*_*i*_(**b**)	*k*_*i*_	σ_*i*_(a)	σ_*i*_(b)
*Of well- balanced love*	1	0.5	0.5	10	0.5	0.5
	2	0.5	0.5	10	0.5	0.5

Let's consider a learning process associated with the structure of the well-balanced love game: the fictitious play process of the well-balanced love game. For *t* = 1, 2, 3, ..., the sequence (p(*t*), q(*t*)) constitutes a discrete fictitious play process (FP) if (p(*t*), q(*t*)) ∈ △(*S*_1_) × △(*S*_2_) such that (p(1), q(1)) is chosen by Nature, and for each *t* = 2, 3, 4, ..., the expectation formation rule described in (12) is satisfied, along with the best response functions of the players as described in Equations 13 and 14.

Consequently, the game *G* is played according to the fictitious play principle if, for each *t*, the condition (Equation 15) is upheld (see Figure 4, Tables 4.B, and 4.C).

**Table 4.B:** Simulation of the game/Fictitious play process.

***t***	**b^1^[*t*]**	**b^2^[*t*]**	**p_1_[*t*]**	**q_1_[*t*]**	***t***	**b^1^[*t*]**	**b^2^[*t*]**	**p_1_[*t*]**	**q_1_[*t*]**
1	0	1	0.8000	0.2000	1	0	1	0.8000	0.0000
2	1	1	1.0000	1.0000	2	1	0	0.0000	1.0000
3	1	1	1.0000	0.5000	3	1	0	0.5000	0.5000
4	0	1	1.0000	0.3333	4	0	1	0.6666	0.3333
5	1	1	1.0000	0.5000	5	1	0	0.5000	0.5000
⋯	⋯	⋯	⋯	⋯	⋯	⋯	⋯	⋯	⋯
996	1	1	0.5005	0.4994	996	0	1	0.5005	0.4994
997	1	0	0.5000	0.5000	997	1	0	0.5000	0.5000
998	0	1	0.5005	0.4994	998	0	1	0.5005	0.4994
999	1	0	0.5000	0.5000	999	1	0	0.5000	0.5000
1,000	0	1	0.5005	0.4994	1,000	0	1	0.5005	0.4994
1	1	1	0.8000	0.8000	1	1	1	0.8000	0.5000
2	1	1	1.0000	1.0000	2	1	1	1.0000	1.0000
3	1	1	1.0000	1.0000	3	1	1	1.0000	1.0000
4	1	1	1.0000	1.0000	4	1	1	1.0000	1.0000
5	1	1	1.0000	1.0000	5	1	1	1.0000	1.0000
⋯	⋯	⋯	⋯	⋯	⋯	⋯	⋯	⋯	⋯
996	1	1	1.0000	1.0000	996	1	1	1.0000	1.0000
997	1	1	1.0000	1.0000	997	1	1	1.0000	1.0000
998	1	1	1.0000	1.0000	998	1	1	1.0000	1.0000
999	1	1	1.0000	1.0000	999	1	1	1.0000	1.0000
1,000	1	1	1.0000	1.0000	1,000	1	1	1.0000	1.0000

The red values indicate the initial conditions (starting points) of the simulation process. These values correspond to the initial probabilities assigned at *t* = 1 before the iterative fictitious play updates.

**Table 4.C:** Simulation of the game/Fictitious play process.

***t***	**b^1^[*t*]**	**b^2^[*t*]**	**p_1_[*t*]**	**q_1_[*t*]**	***t***	**b^1^[*t*]**	**b^2^[*t*]**	**p_1_[*t*]**	**q_1_[*t*]**
1	0	0	0.2000	0.2000	1	0	0	0.5000	0.2000
2	0	0	0.0000	0.0000	2	0	0	0.0000	0.0000
3	0	0	0.0000	0.0000	3	0	0	0.0000	0.0000
4	0	0	0.0000	0.0000	4	0	0	0.0000	0.0000
5	0	0	0.0000	0.0000	5	0	0	0.0000	0.0000
⋯	⋯	⋯	⋯	⋯	⋯	⋯	⋯	⋯	⋯
996	0	0	0.0000	0.0000	996	0	0	0.0000	0.0000
997	0	0	0.0000	0.0000	997	0	0	0.0000	0.0000
998	0	0	0.0000	0.0000	998	0	0	0.0000	0.0000
999	0	0	0.0000	0.0000	999	0	0	0.0000	0.0000
1000	0	0	0.0000	0.0000	1000	0	0	0.0000	0.0000
1	1	0	0.2000	0.8000	1	1	0	0.2000	0.5000
2	0	1	1.0000	0.0000	2	0	1	1.0000	0.0000
3	1	0	0.5000	0.5000	3	1	0	0.5000	0.5000
4	0	1	0.6600	0.3300	4	0	1	0.6600	0.3300
5	1	0	0.5000	0.5000	5	1	0	0.5000	0.5000
⋯	⋯	⋯	⋯	⋯	⋯	⋯	⋯	⋯	⋯
996	1	0	0.5005	0.4994	996	1	0	0.5005	0.4994
997	0	1	0.5000	0.5000	997	0	1	0.5000	0.5000
998	1	0	0.5005	0.4994	998	1	0	0.5005	0.4994
999	0	1	0.5000	0.5000	999	0	1	0.5000	0.5000
1,000	1	0	0.5005	0.4994	1,000	1	0	0.5005	0.4994

The red values indicate the initial conditions (starting points) of the simulation process. These values correspond to the initial probabilities assigned at *t* = 1 before the iterative fictitious play updates.

Corollary 6. *Of well-balanced love*—. If Eduard and Alice recognize each other as equals and share the same preferences, then the woman and man in the Nash equilibrium will meet and stay together. Formally, if *u*_*i*_(a) = *u*_*i*_(b) = 1/2 for each *i* = 1, 2 then


$$\left(\text{p*},\text{q*}\right)=\left(\left(p^{*},1-p^{*}\right),\left(q^{*},1-q^{*}\right)\right)=\left(\left(1/2,1/2\right),\left(1/2,1/2\right)\right)$$

is the unique completely mixed equilibrium of the game *G* (see Figure 4).

#### 3.4.2 Social choice and power structures in well-balanced love

In a “balanced love” that does not reproduce the power structures of “courtly love,” “patriarchal love,” or “hedonistic love,” its associated power structure can be described as more equitable and just. The preferences and levels of empathy described by the balanced love model result in an expression of shared reciprocity and empathy, as empathy is not unbalanced as it is in patriarchal, hedonistic, or courtly love models. Instead of a one-sided exchange of empathy, in balanced love, both parties show and receive empathy in an equitable but moderate manner, fostering a deep and meaningful emotional connection. This allows us to assert that the balanced love model reflects the autonomy and agency that each individual exercises within the relationship. Individual and joint decisions are made collaboratively, respecting each other's independence and personal desires. Consequently, the balanced love model describes a power structure where equity, reciprocity, and mutual respect are fundamental. The social choice in a balanced love is based on norms that promote equality and fairness between the parties. Social and cultural decisions reflect a rejection of traditional power hierarchies and an acceptance of relationships where both individuals are seen and treated as equals. The balanced love model demonstrates that, under given conditions, the equilibrium is one of stable, enduring love.

### 3.5 Simplifying assumptions and external validity

The proposed theoretical model is built upon a series of simplifying assumptions that, while enabling a rigorous formalization of strategic equilibria in romantic relationships, also constrain the external validity of the results. Below, we discuss the most relevant assumptions and their implications for the empirical applicability of the model.

(1) **Binary project space**. The model assumes that each individual chooses between two alternative life projects, a and b. This simplification allows for a clear representation of structural tensions—for example, between professional career and family, or between sexuality and spirituality—where the involved trajectories are hardly compatible in terms of time, resources, affective commitments, or identity formation. Although this binary reduction omits the multidimensional and complex nature of life projects in real-world contexts, it follows an analogous logic to that used in microeconomic consumer theory, where two goods are modeled to graphically illustrate preferences and constraints. Beyond its representational value, the use of two mutually exclusive projects constitutes a substantive modeling decision: it captures structural heterogeneity in individual preferences with the minimal level of necessary differentiation. In this way, strategic discoordination between agents does not emerge from misunderstandings or miscalculations but from a real tension between life visions whose joint realization is structurally limited. Nevertheless, this binary structure restricts the analysis to dichotomous dilemmas, which may limit the representativeness of affective trajectories in more fluid contexts or those involving partially reconcilable projects.

(2) **Constant and culturally determined empathy**. The empathy coefficient *k*_*i*_ is treated in the model as a constant and exogenous parameter throughout the strategic interaction. This constancy does not imply that empathy is innate or natural, nor does it exclude its origin in processes of affective socialization, normative learning, or cultural internalization. On the contrary, the model assumes that *k*_*i*_ represents an affective disposition historically shaped by cultural norms, symbolic traditions, and relational archetypes. Thus, an individual raised in a culture that promotes unilateral sacrifice may exhibit very low or very high values of *k*_*i*_, depending on what has been internalized as legitimate love. The assumption of constancy refers solely to the fact that this parameter does not change within the framework of the game once it has been established; that is, it does not adjust endogenously in response to the strategic dynamics. Although the model does not incorporate intra-relational mechanisms for empathy change, it does acknowledge its structurally produced and relatively stable nature within specific historical contexts.

(3) **Formal symmetry with substantive asymmetry**. Although the model assumes a formally symmetric structure—both players share an identical set of strategies and a utility function with the same architecture—this structural symmetry does not entail substantive equality between agents. On the contrary, the model allows for, and is indeed designed to represent, deeply asymmetric relationships, such as those associated with gender inequalities, through the differentiated assignment of empathy coefficients *k*_*i*_. These coefficients embody internalized affective dispositions derived from processes of cultural socialization, enabling the model to distinguish between archetypes such as patriarchal, courtly, or hedonistic love. In this way, asymmetry in empathy makes it possible to formalize power and affective structures in strategic terms, avoiding a neutral idealization of the romantic bond and emphasizing its normative and conflictual dimension. In this sense, the model does not bypass inequalities: it integrates them as central parameters of strategic behavior.

(4) **Static game with complete information**. The baseline structure of the game is static and unfolds under conditions of complete information; that is, each player knows the preferences and empathy levels of the other. This assumption is methodologically convenient for identifying equilibria, but it does not reflect real-world situations in which the motivations of the other are partially unknown, ambiguous, or subject to strategic revelation. Although the model is extended through fictitious play to incorporate an iterative dynamic, the structure of belief formation and learning remains limited when compared to Bayesian models or settings with imperfect information.

These assumptions are common in formal models within the social sciences and serve a heuristic function by enabling the isolation of causal mechanisms and the analysis of interaction patterns. However, their simplification entails that the results should be interpreted as theoretical propositions about possible logics of affective interaction, rather than as direct empirical descriptions of specific cases. The model's external validity, therefore, depends on its use as a tool of theoretical idealization—one that can generate hypotheses, scenarios, and future tests, both quantitative and qualitative.

## 4 Final notes

This article provides theoretical elements that contribute to addressing an unresolved scientific issue related to the lack of a comprehensive and rigorous characterization of the irrationality of love from a rationality perspective in family literature. While theoretical models have been constructed to understand human interactions traditionally considered irrational, such as love, there is currently no characterization of this irrationality from a rationality approach in the existing family literature. This article, with the proposed methodological approach, offers the development and application of solid theoretical and methodological models that allow for a deeper and more complete understanding of the irrationality of love in the context of family interactions, enabling future research to address questions such as: How can theoretical models be designed to capture the complexity and variety of cultural conceptions of love within family dynamics? What factors influence the formation and evolution of different conceptions of love within family structures? How can instrumental rationality be integrated into understanding behaviors related to love and their impact on family relationships? What are the practical and ethical implications of applying rationality approaches to issues related to love and family, especially in terms of therapeutic interventions or family policies? What research methods and techniques may be most effective for exploring and analyzing the irrationality of love in specific family contexts, taking into account cultural diversity and individual differences?

The social choice and power structures behind individual preferences and their empathy coefficients satisfactorily explain the dynamics of pairings in contexts that are already considered classic references. The proposed models constitute a novel contribution to the literature.

## Data Availability

The datasets generated through the computational simulations described in the article are available from the corresponding author upon reasonable request.

## Footnotes

^1^During the data collection, processing, and presentation phases of the research, the VOSviewer application was utilized. Additionally, in the assessment of scientific activity, bibliometric measures were applied to analyze productivity, collaboration, and detect the coexistence of key terms. Considering the 1,080 documents found with the search equation Love AND Empathy, it is observed that since the year 2000, the number of published articles studying these two topics has been increasing. The highest production is recorded in the years 2020, 2021, and 2022, with 86, 80, and 90 articles published, respectively, representing 24% of the total. The analysis of the distribution of research on Love AND Empathy reveals a wide diversity of academic fields interested in these topics. Medicine accounts for 21% of the publications, and the social sciences, with nearly 20%, also show significant interest in empathy and love. This reflects the importance of these concepts in understanding social dynamics, interpersonal relationships, and human behavior in general. The fact that 18% of the research comes from the arts, humanities, and psychology underscores the emotional and subjective dimension of empathy and love. These disciplines can provide deeper insights into the human experience and how these feelings manifest in culture, art, and individual psychology. Collectively, this analysis highlights the breadth of interest in empathy and love across various fields of knowledge. The growing interest from medicine, social sciences, arts, and psychology in these topics demonstrates the increasing significance of these aspects in human life and their relevance in decision-making in fields such as healthcare, psychotherapy, and the cultivation of more empathetic and loving societies. Based on the information above, a node analysis was conducted using the VOSviewer tool. The main thematic axes addressed by the scientific community are directly related to empathy, love, caregiving, ethics, morality, and affection, which have captured the attention of researchers from various disciplines, ranging from psychology and neuroscience to philosophy and sociology.

^2^In particular, in India and Pakistan, marriages are arranged due to the belief that parents or elders in the community have a greater ability to choose a compatible partner in terms of cultural, religious, social, and economic values. However, arranged marriages do not imply that they are imposed, as individuals have the capacity to accept or reject marriage proposals, and their consent is a fundamental aspect. Generally, for each cultural identity regarding love, there exists a prototype interaction mechanism between two individuals with certain inclinations toward reciprocity and empathy in their relationships. Therefore, the proposed theoretical model is not a zero-sum game.

^3^There are behaviors such as neurosis that, despite being a behavioral disorder, can be explained from an instrumental rationality perspective. Neurosis, as a behavioral disorder, disrupts an individual's rational thinking. Both love and neurosis can exhibit illogical behaviors that challenge conventional notions of instrumental rationality. The debate about whether individuals consciously and deliberately adopt neurosis as a behavioral strategy is not relevant from a methodological standpoint. Methodologically, it is argued that individuals adopt neurosis as a rational response to the cognitive and emotional complexities of their environment. The adoption of neurosis is an instrumental response by the individual to make their psycho-affective system function effectively. However, this does not imply that individuals consciously employ logical-deductive reasoning to determine the adoption of specific neurotic behaviors, such as “hysterical blindness” or compulsive thumb-sucking. While it is evident that neurosis as behavior is irrational, from a methodological perspective, the adoption of neurosis as a response to an overwhelming environment can be considered rational. In this sense, neurosis can be understood as a manifestation of an individual's psycho-affective system response to challenges presented by their environment.
